# First insights on the susceptibility of native coccidicidal fungi *Mucor circinelloides* and *Mucor lusitanicus* to different avian antiparasitic drugs

**DOI:** 10.1186/s12917-024-03909-z

**Published:** 2024-02-23

**Authors:** João Lozano, Eva Cunha, Luís Madeira de Carvalho, Adolfo Paz-Silva, Manuela Oliveira

**Affiliations:** 1 CIISA – Centre for Interdisciplinary Research in Animal Health, Faculty of Veterinary Medicine, University of Lisbon, Avenida da Universidade Técnica, Lisbon, 1300-477 Portugal; 2 Associate Laboratory for Animal and Veterinary Sciences (AL4AnimalS), Lisbon, 1300-477 Portugal; 3 Control of Parasites Research Group (COPAR, GI-2120), Department of Animal Pathology, Faculty of Veterinary, University of Santiago de Compostela, Lugo, 27142 Spain; 4 cE3c – Centre for Ecology, Evolution and Environmental Changes & CHANGE – Global Change and Sustainability Institute, Faculdade de Ciências, Universidade de Lisboa, Lisbon, 1749-016 Portugal

**Keywords:** Gastrointestinal parasites, Predatory fungi, *Mucor* spp., Antiparasitic drugs, Susceptibility

## Abstract

**Background:**

The combined application of predatory fungi and antiparasitic drugs is a sustainable approach for the integrated control of animal gastrointestinal (GI) parasites. However, literature addressing the possible interference of antiparasitic drugs on the performance of these fungi is still scarce. This research aimed to assess the in vitro susceptibility of six native coccidicidal fungi isolates of the species *Mucor circinelloides* and one *Mucor lusitanicus* isolate to several antiparasitic drugs commonly used to treat GI parasites’ infections in birds, namely anthelminthics such as Albendazole, Fenbendazole, Levamisole and Ivermectin, and anticoccidials such as Lasalocid, Amprolium and Toltrazuril (drug concentrations of 0.0078–4 µg/mL), using 96-well microplates filled with RPMI 1640 medium, and also on Sabouraud Agar (SA).

**Results:**

This research revealed that the exposition of all *Mucor* isolates to the tested anthelminthic and anticoccidial drug concentrations did not inhibit their growth. Fungal growth was recorded in RPMI medium, after 48 h of drug exposure, as well as on SA medium after exposure to the maximum drug concentration.

**Conclusions:**

Preliminary findings from this research suggest the potential compatibility of these *Mucor* isolates with antiparasitic drugs for the integrated control of avian intestinal parasites. However, further in vitro and in vivo studies are needed to confirm this hypothesis.

## Background

Domestic, exotic, and wild birds kept in captivity often contact with the same outdoor area for long periods of time, and thus are highly prone to re-infections caused by the infective forms of several gastrointestinal (GI) parasites, namely coccidia and helminths, which are responsible for clinical or sub-clinical diseases, and major economic concerns in poultry farms, zoological parks, and private bird collections [1–4].

Their prevention and treatment are still frequently performed exclusively with antiparasitic drugs, namely coccidiostatics (e.g., Amprolium and Lasalocid), coccidicidals (e.g., Toltrazuril) and anthelminthics (e.g., Benzimidazoles and Macrocyclic Lactones), whose incorrect use often leads to antiparasitic drug resistance, and accumulation of drug residues in bird carcasses, soil, and ground-waters [5–8].

Since the early 1990’s, researchers from around the world have been proposing the integration of predatory fungi in animal health programs in farms, zoos, and private animal collections, serving as a complement to antiparasitic drugs for the control of GI parasitic infections in domestic, companion, exotic and captive wild animals [9–12].

The main attribute of these fungi lays on their ability to destroy parasites’ infective stages (oocysts, eggs, or larvae), and thus breaking their life cycles in the environment. The most frequently reported predatory fungal taxa are: *Duddingtonia flagrans*, *Arthrobotrys* spp. and *Monacrosporium* spp., which are larvicidal fungi and thus predate and destroy nematodes’ infective larvae (L3); *Pochonia chlamydosporia*, *Mucor circinelloides* and *Verticillium* spp., which have shown to present ovicidal properties, destroying both nematodes’ eggs and coccidia oocysts [9, 10, 13–16].

Considering that fungi of the order Mucorales are commonly associated to opportunistic infections [17, 18], ensuring their safety for animals is a mandatory step while designing a parasite biocontrol program, namely through anatomopathological, cytotoxicity, hematological and fecal analysis. In fact, all previous studies revealed that parasitized animals receiving *M. circinelloides* spores maintained or even improved the hematological parameters and feces consistency and appearance, and also without damaging the internal tissues [19–22]. Predatory fungi are administrated to animals always in controlled programs, with constant monitorization of any side effects [15, 22, 23].

Moreover, ensuring their environmental innocuity is essential, namely to free-living nematodes which have an important role in soil and plant roots’ oxygenation. For this purpose, a previous study developed by Saumell et al. [24] demonstrated that the presence of *D. flagrans* spores in ovine feces does not have any effect on its natural colonization by free-living nematodes and other native predatory fungi, and thus not posing any environmental concern.

Combining antiparasitic drug treatments with predatory fungi administrations is of major importance, to target parasites’ endogenous and exogenous stages [9, 23]. However, information regarding the possible negative effect of antiparasitic drugs in the survival of predatory fungi spores is still scarce, being a critical step in the design of an integrated parasite control program. Previous in vitro and in vivo research performed with the larvicidal fungi *D. flagrans* and *Arthrobotrys* spp., and the ovicidal fungi *Paecilomyces* spp. and *Verticillium chlamydosporium* (furtherly reclassified as *P. chlamydosporia*), have revealed these fungal taxa as being susceptible to variable concentrations of Ivermectin and several Benzimidazoles [25–28]. However, there is little information on the possible susceptibility of other predatory fungi taxa to different anticoccidial and anthelmintic drugs.

The current research aimed to assess for the first time the potential susceptibility of seven native ovicidal fungi of the genus *Mucor* to different antiparasitic drugs commonly used in Avian Medicine.

## Results

**Fig. 1:**
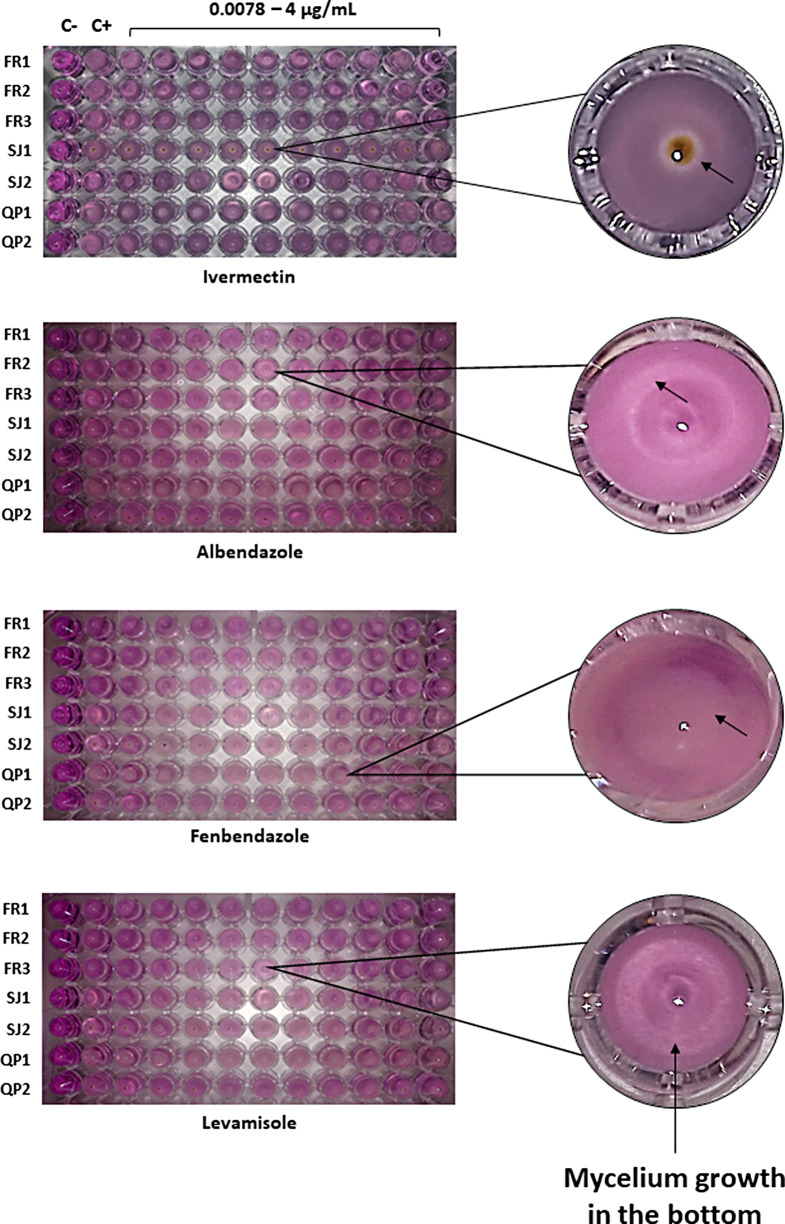
Fungal growth recorded for each *Mucor* isolate after exposition to each anthelminthic drug concentration. Legend FR1 – *M. circinelloides* isolate FMV-FR1; FR2 – *M. circinelloides* isolate FMV-FR2; FR3 – *M. circinelloides* isolate FMV-FR3; SJ1 – *M. circinelloides* isolate FMV-SJ1; SJ2 – *M. circinelloides* isolate FMV-SJ2; QP1 – *M. lusitanicus* isolate FMV-QP1; QP2 – *M. circinelloides* isolate FMV-QP2; C+:positive control (medium and fungi), C-:negative control (only medium); magnifications for each drug plate illustrate examples of growth detected in those wells (black arrows)

**Fig. 2:**
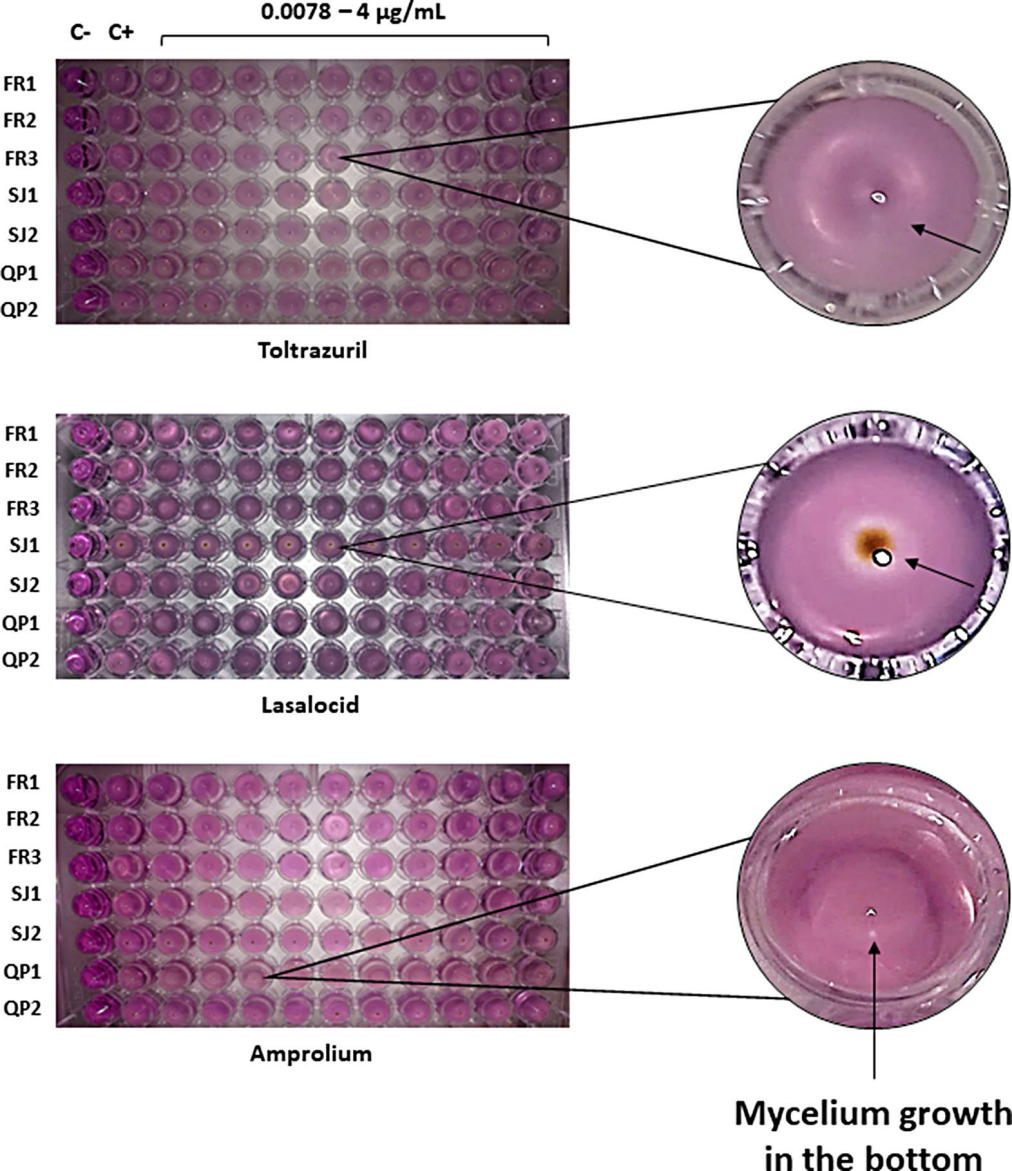
Fungal growth recorded for each *Mucor* isolate after exposition to each anticoccidial drug concentration. Legend FR1 – *M. circinelloides* isolate FMV-FR1; FR2 – *M. circinelloides* isolate FMV-FR2; FR3 – *M. circinelloides* isolate FMV-FR3; SJ1 – *M. circinelloides* isolate FMV-SJ1; SJ2 – *M. circinelloides* isolate FMV-SJ2; QP1 – *M. lusitanicus* isolate FMV-QP1; QP2 – *M. circinelloides* isolate FMV-QP2; C+:positive control (medium and fungi), C-:negative control (only medium); magnifications for each drug plate illustrate examples of growth detected in those wells (black arrows)

Antiparasitic drug susceptibility experiments revealed that all seven *Mucor* isolates were not susceptible to the antiparasitic drugs tested and for all their assessed concentrations. Fungal growth was observed after two days of incubation, as demonstrated by the detection of mycelium growth in the bottom of test and positive control wells. These results reveal that spores’ survival and mycelium growth were not affected by the exposure to antiparasitic drugs. Also, no fungal growth was recorded in the negative control, and thus confirming no contamination during the assay (Figs. 1 and 2).

**Fig. 3:**
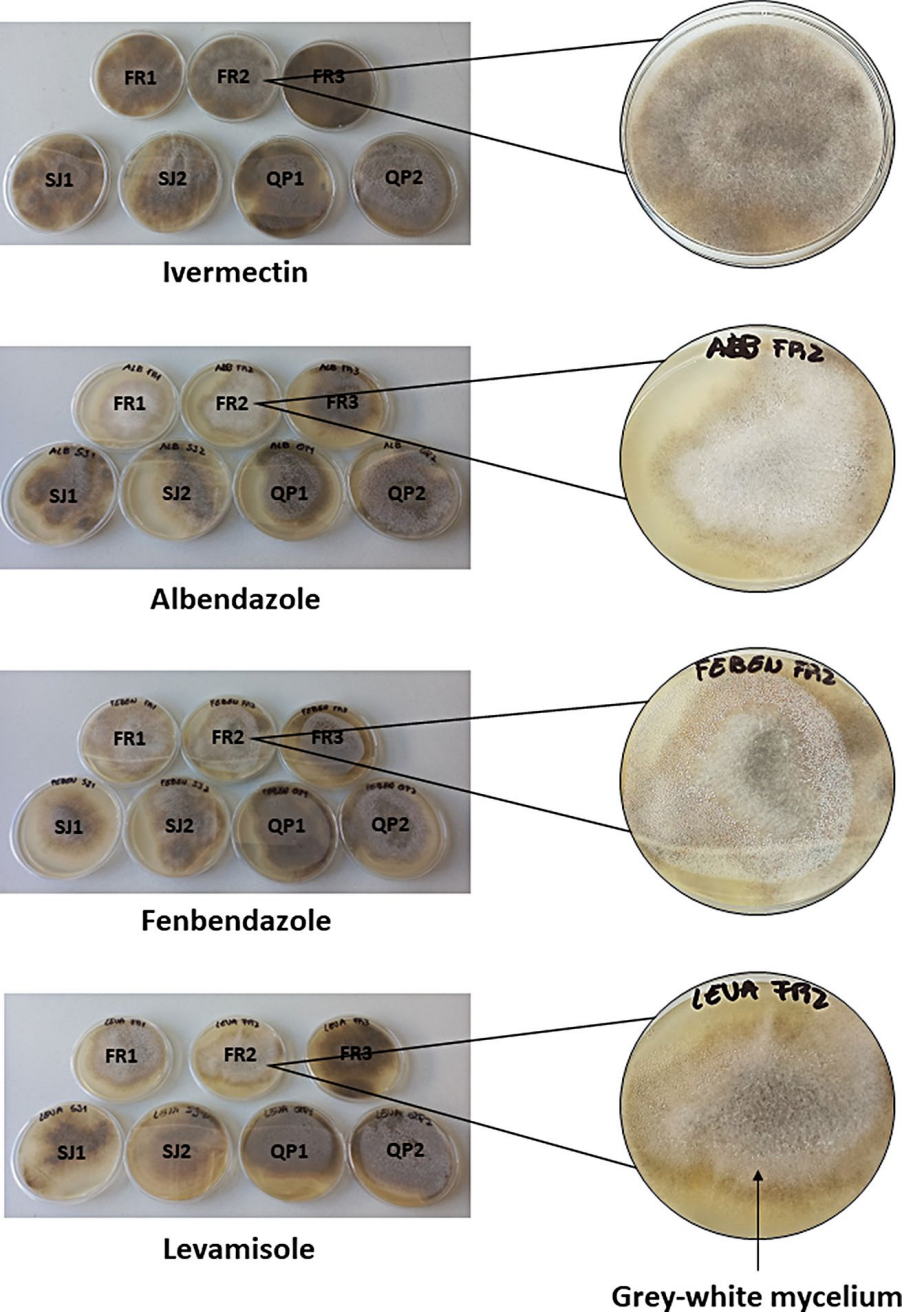
Fungi germination on Sabouraud Agar, after exposition to the maximum anthelminthic concentration of 4 µg/mL. Legend FR1 – *M. circinelloides* isolate FMV-FR1; FR2 – *M. circinelloides* isolate FMV-FR2; FR3 – *M. circinelloides* isolate FMV-FR3; SJ1 – *M. circinelloides* isolate FMV-SJ1; SJ2 – *M. circinelloides* isolate FMV-SJ2; QP1 – *M. lusitanicus* isolate FMV-QP1; QP2 – *M. circinelloides* isolate FMV-QP2; magnifications provide an insight on the growth of typical *Mucor* spp. colonies on Sabouraud agar (black arrow)

**Fig. 4:**
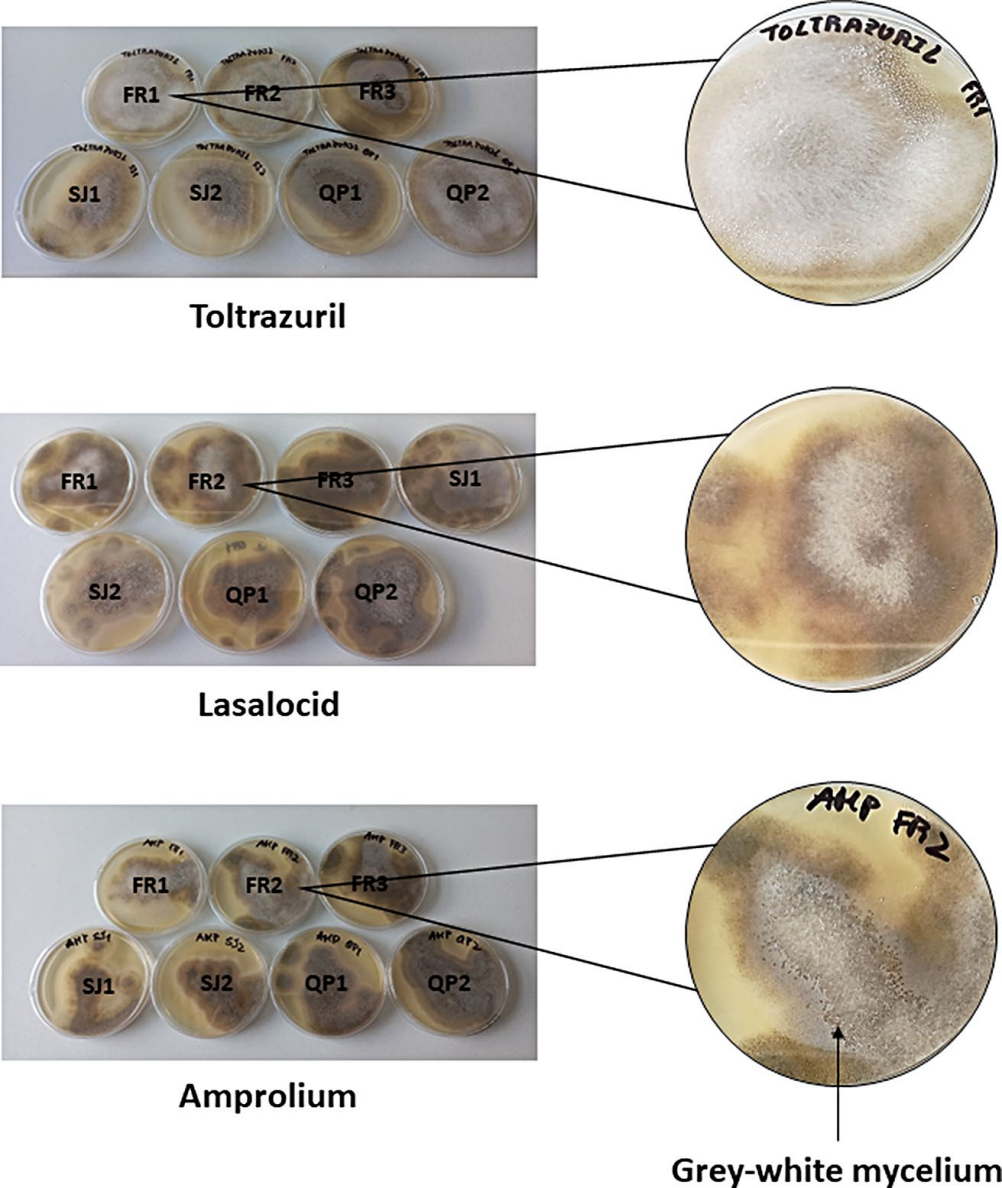
Fungi germination on Sabouraud Agar, after exposition to the maximum anticoccidial concentration of 4 µg/mL. Legend FR1 – *M. circinelloides* isolate FMV-FR1; FR2 – *M. circinelloides* isolate FMV-FR2; FR3 – *M. circinelloides* isolate FMV-FR3; SJ1 – *M. circinelloides* isolate FMV-SJ1; SJ2 – *M. circinelloides* isolate FMV-SJ2; QP1 – *M. lusitanicus* isolate FMV-QP1; QP2 – *M. circinelloides* isolate FMV-QP2; magnifications provide an insight on the growth of typical *Mucor* spp. colonies on Sabouraud agar (black arrow)

Moreover, all fungal isolates maintained their germination capacity even after exposition to the maximum drug concentration of 4 µg/mL, for all anticoccidials and anthelmintics, as demonstrated by the macroscopical visualization of colonies growth on SA medium (Figs. 3 and 4).

## Discussion

Assessing predatory fungi susceptibility to antiparasitic drugs is an important step in the optimization of parasite biocontrol programs, and despite fungi are not exposed to the initial drug concentration administrated to animals, in the intestinal microenvironment, any fungal incompatibility to a given drug might interfere with its germination capacity, and further efficacy on destroying parasitic forms [25–28].

Results obtained in this research revealed that all studied *Mucor* isolates were not susceptible to anticoccidial and anthelminthic drugs, independently of the drug concentration, maintaining their germination capacity after drug exposure. These results are in contrast with previous research performed in vitro with larvicidal and ovicidal fungi [27, 28]. In vitro susceptibility of predatory fungal species to antiparasitic drugs was first described by Ferreira et al. [27], who reported that Ivermectin and Albendazole concentrations as low as 0.08 mg/mL had an inhibitory effect on *Paecilomyces* spp. growth of approximately 11–63% and 60–79%, respectively. Also, another study performed by Vieira et al. [28], revealed that Albendazole, Thiabendazole, Ivermectin, Levamisole and Closantel had an inhibitory effect on the growth of *D. flagrans*, *Arthrobotrys oligospora*, and *P. lilacinus*. Thus, it can be concluded that for a given predatory fungus strain, a previous in vitro assessment of antiparasitic drug susceptibility might be of most importance to establish the optimal combination of drug treatment and fungal administration.

Also, it is of major importance that studies on this topic standardize the protocol used for assessing fungal susceptibility to different antiparasitic drugs, by following the international guidelines established by the Clinical & Laboratory Standards Institute (CLSI), for filamentous fungi [29], as performed in our study, or by the European Committee on Antimicrobial Susceptibility Testing (EUCAST), which is also another worldwide recognized organization in the field of medical testing.

A possible explanation for the lack of susceptibility of all *Mucor* isolates to the tested antiparasitic drugs, namely to Benzimidazoles, might rely on the incapacity of these drugs to bind to its β-tubulins. These proteins are essential for microtubules’ stabilization during the interphase stage of the fungal cells’ cycle, and it has been demonstrated that Benzimidazoles’ binding to these proteins arrests fungal cell division [25, 30, 31]. A systematic identification of tubulin genes from 59 representative fungi reported that *M. circinelloides* has four β-tubulin genes [32], and therefore any mutation on these genes might interfere with the drugs’ binding sites, as previously demonstrated for different fungal taxa [33]. However, further studies are needed to confirm this hypothesis, namely through Whole-Genome Sequencing (WGS) of the tested *Mucor* isolates.

Our study followed the standard M38 established by CLSI, which is one of the leading worldwide organizations to provide guidelines and standards in medical laboratory testing. The used standard only mentions a qualitative analysis for assessing the susceptibility of fungi to drugs, which was enough in the current study to check if fungi isolates were capable of growing during and after drug exposition. Moreover, the current study also included some relevant modifications of the CLSI standard M38, which only established the application of a negative control well (with only RPMI medium), considering it as sufficient for this kind of antimicrobial susceptibility assays, and just for testing contamination. Our team complemented the assays by including a positive control (fungi and RPMI), to perform a more robust analysis, and check if fungal isolates were capable of growing in RPMI when not exposed to antiparasitic drugs, as described also by Vieira et al. [28]. Besides, two trials were performed (in microplates and SA medium), to confirm the lack of susceptibility of all *Mucor* isolates to the different antiparasitic drugs, by checking if fungi were capable of growing on SA medium following exposition to the maximum drug concentration (4 µg/mL). Although a qualitative analysis is enough to assess if a certain fungal isolate is susceptible to a given antimicrobial drug, it would be also interesting if further studies in this topic include a quantitative analysis, namely the quantification of Colony Forming Units (CFUs) and each well’s absorbance, as well as measuring colony radial growth in different timepoints following drug exposition. Moreover, and despite predatory fungal spores and drugs are often administrated separately to animals in different timepoints, with an interval of 14–21 days post-drug treatment [20, 34, 35], and thus fungi are not exposed to the initial drug dosage, but instead to lower concentrations due to its metabolization in the gastrointestinal tract, it would be interesting in further studies to assess if these native *Mucor* isolates maintain their lack of susceptibility to several therapeutic dosages used for the tested antiparasitic drugs, and thus conclude if a drug wash-out period is necessary before administrating fungal spores.

## Conclusion

To our best knowledge, this is the first report to reveal the compatibility of different isolates of the genus *Mucor* to the most common avian antiparasitic drugs, and thus suggesting these parasiticide fungi as potential candidates to be combined with the most common anticoccidials and anthelmintics in integrated parasite biocontrol programs in domestic and exotic bird collections. However, further in vitro and in vivo studies are needed and in current progress to confirm these hypotheses.

## Methods

A total of seven *Mucor* isolates of the species *M. circinelloides* (FMV-FR1, FMV-FR2, FMV-FR3, FMV-SJ1, FMV-SJ2, FMV-QP2) and *Mucor lusitanicus* (FMV-QP1), belonging to the native predatory fungi collection of the Parasitology and Parasitic Diseases Lab, Faculty of Veterinary Medicine - University of Lisbon, and with proven parasiticide activity towards avian coccidia [14], were used in this research. All fungal isolates were previously obtained from chicken and peacock fecal samples, and subjected to morphological and molecular identification through amplification of rDNA’s ITS1-5.8 S-ITS2 region and further sequencing using the ITS1 primer [14]. Moreover, isolates were maintained in Wheat-Flour Agar (WFA, 2%), at room temperature, as previous research revealed this medium to be a good alternative to other more nutritive mediums like Corn Meal, Potato Dextrose or Malt Extract agar, for rapid hyphae growth and storage of purified ovicidal fungi cultures [14, 16].

Fresh mycelium was collected from each fungal isolate, using a calibrated 1 µL swab, and diluted in distilled water, with spores’ final concentration being calculated using the Neubauer chamber. Fungal concentrations were standardized to 10^6^ spores/mL.

All fungal isolates were checked against several antiparasitic drugs commonly used to treat coccidia and helminth infections in birds, namely Ivermectin (Purity ≥ 90%, Molecular Weight (MW) = 875.1 g/mol, Solubility in DMSO = 50 mg/mL; Merck Life Science, S.L., Lisbon, Portugal), Lasalocid (Purity ≥ 97%, MW = 612.77 g/mol, Solubility = 100 mg/mL; Ehrenstorfer GmbH, Augsburg, Germany), Albendazole (Purity ≥ 98%, MW = 265.33 g/mol, Solubility = 17 mg/mL; Merck Life Science, S.L., Lisbon, Portugal), Amprolium (Purity ≥ 98%, MW = 315.24 g/mol, Solubility = 2 mg/mL; Merck Life Science, S.L., Lisbon, Portugal), Toltrazuril (Purity ≥ 98%, MW = 425.38 g/mol, Solubility = 25 mg/mL; Merck Life Science, S.L., Lisbon, Portugal), Fenbendazole (Purity ≥ 98%, MW = 299.35 g/mol, Solubility = 30 mg/mL; Merck Life Science, S.L., Lisbon, Portugal) and Levamisole (Purity ≥ 98%, MW = 240.75 g/mol, Solubility = 10 mg/mL; Merck Life Science, S.L., Lisbon, Portugal).

Techniques used in this assay were based on the international standards proposed by CLSI for assessing filamentous fungi susceptibility to antimicrobial drugs [29], as well as in previous research with larvicidal and ovicidal fungi [28].

Stock solutions of each antiparasitic drug were prepared according to the manufacturers’ instructions and each drug’s solubility, having all been dissolved in Dimethyl Sulfoxide medium (DMSO) (Avantor, Inc., Radnor, Pennsylvania, USA) to a concentration of 1 mg/mL (5 mg of each drug diluted in 5 mL of DMSO). Working solutions of 100 µg/mL were prepared for each drug using also DMSO (100 µL of stock solution diluted in 900 µL of DMSO), followed by dilution in Roswell Park Memorial Institute medium (RPMI 1640) (Biowest, Missouri, USA) to a concentration of 8 µg/mL (80 µL of working solution diluted in 920 µL of RPMI).

Serial dilutions (1:2) were performed in 96-well microplates, using drug concentrations ranging between 0.0078 and 4 µg/mL, with wells containing a final fungal concentration of 10^5^ spores/mL (100 µL of each fungus and 100 µL of each drug concentration). Positive and negative controls (100 µL of each fungus and 100 µL of RPMI medium, and only 200 µL of RPMI, respectively) were also used to test fungal growth in RPMI medium and contamination, respectively. Plates were incubated at 26 °C for 48 h, using a compressor-cooled incubator. Three independent assays were performed, using two replicates for each fungal isolate and drug. After incubation, each well’s bottom was checked for mycelia growth, by directly visualization (naked eye), with two possible outcomes: the lack of mycelia in the bottom of the test wells means a fungistatic effect of the respective drug concentration, whereas the growth of fungal mycelia means that the isolate is not susceptive.

**Fig. 5:**
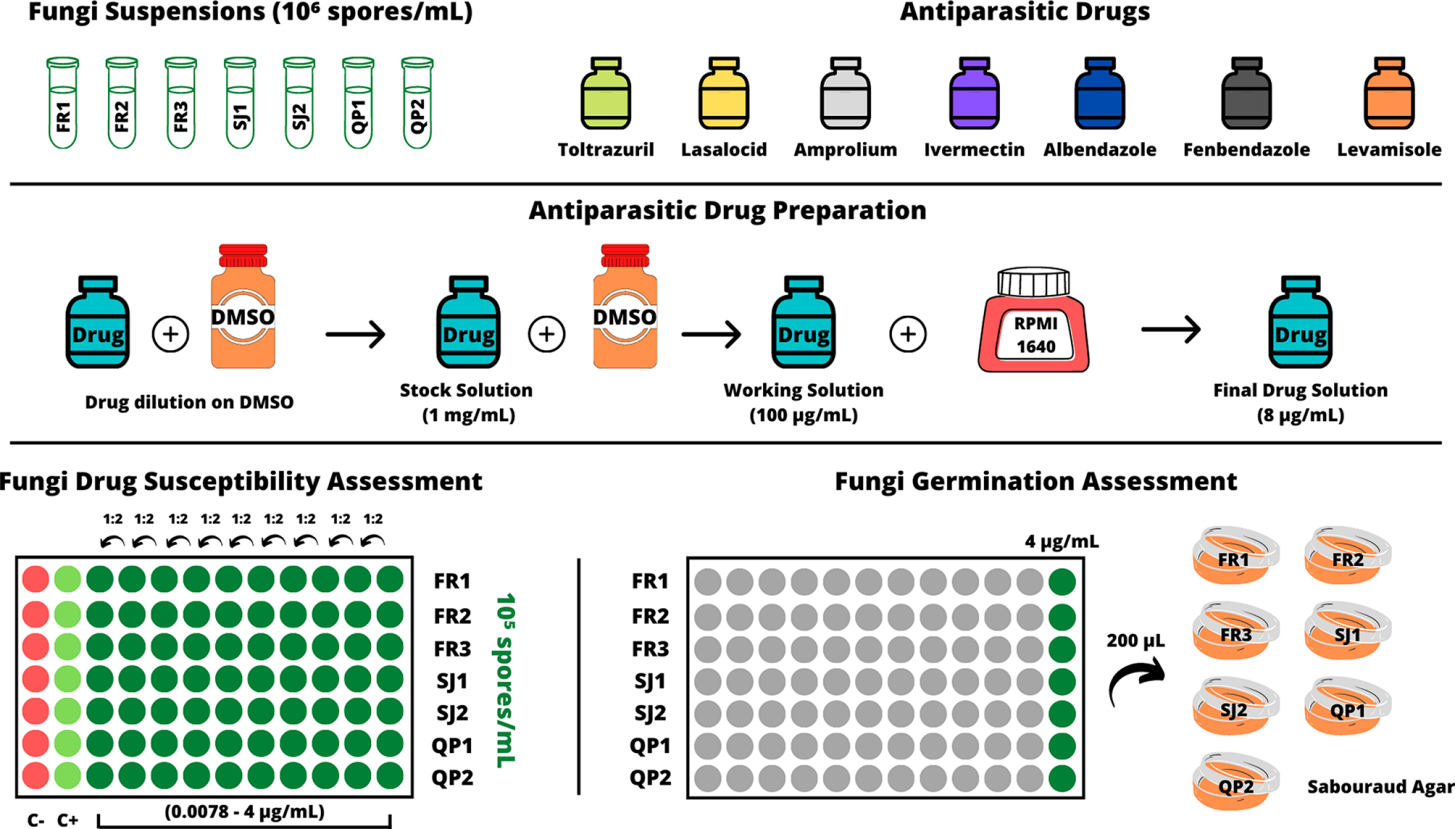
Workflow followed in the current study (figure created using Canva®; www.canva.com). Legend FR1 – *M. circinelloides* isolate FMV-FR1; FR2 – *M. circinelloides* isolate FMV-FR2; FR3 – *M. circinelloides* isolate FMV-FR3; SJ1 – *M. circinelloides* isolate FMV-SJ1; SJ2 – *M. circinelloides* isolate FMV-SJ2; QP1 – *M. lusitanicus* isolate FMV-QP1; QP2 – *M. circinelloides* isolate FMV-QP2

The total suspension (200 µL) in wells containing the highest drug concentration (4 µg/mL) was finally transferred to Sabouraud Agar (SA), having these plates been also incubated at 26 °C for 48 h, to check for the maintenance of fungal growth after exposure to the respective antiparasitic drug, and with also two possible outcomes: the lack of fungal growth on this medium means a fungicide effect promoted by the corresponding drug, whereas colony growth means that the isolate was not susceptive to the corresponding drug. Both approaches were used for all fungal isolates, even if fungal growth was recorded in all test wells, to counter any dubious mycelia growth result, and therefore using the assay on SA medium as the final proof for any fungal susceptibility to antiparasitic drugs (Fig. 5).

The chosen drug concentration range of 0.0078–4 µg/mL was based on: (i) Vieira et al. [28], who used drug concentrations of 0.0078–4 µg/mL for albendazole, thiabendazole and ivermectin, 0.003–1.875 µg/mL for levamisole, and 0.004–2.5 µg/mL for closantel, and reported Minimum Inhibitory Concentrations (MIC’s) as low as 0.031–4 µg/mL for *A. oligospora*, *D. flagrans* and *P. lilacinus* (furtherly reclassified as *Purpureocillium lilacinum*); (ii) Sanyal et al. [25] and Singh et al. [26] studies, who reported fungal growth inhibitions at Albendazole and Triclabendazole concentrations of 1–4.5 µg/mL, after spores being fed to Ruminants; (iii) therapeutic dosages of ivermectin as low as 0.8–1 µg/mL (drinking-water) in some exotic birds species, namely canaries [36]. This information was used as a starting point for establishing the drug range in the current research, and find which drugs are compatible with the used native fungal isolates.

## Data Availability

All data generated or analysed during this study are included in this published article.

## Abbreviations

GI: Gastrointestinal
SA: Sabouraud Agar
L3: Infective Larvae
CLSI: Clinical & Laboratory Standards Institute
EUCAST: European Committee on Antimicrobial Susceptibility Testing
WGS: Whole-Genome Sequencing
CFUs: Colony Forming Units
WFA: Wheat-Flour Agar
MW: Molecular Weight
DMSO: Dimethyl Sulfoxide
RPMI 1640: Roswell Park Memorial Institute medium
